# Differential normal skin transcriptomic response in total body irradiated mice exposed to scattered versus scanned proton beams

**DOI:** 10.1038/s41598-021-85394-0

**Published:** 2021-03-12

**Authors:** Alexandre Leduc, Samia Chaouni, Frédéric Pouzoulet, Ludovic De Marzi, Frédérique Megnin-Chanet, Erwan Corre, Dinu Stefan, Jean-Louis Habrand, François Sichel, Carine Laurent

**Affiliations:** 1 Normandie Univ, UNICAEN, UNIROUEN, ABTE-EA4651, ToxEMAC, Cancer Centre François Baclesse, 14000 Caen, France; 2 Institut Curie, RadeXp Platform, centre universitaire, 91405 Orsay, France; 3 Institut Curie, PSL Research University, University Paris Saclay, Laboratoire d’Imagerie Translationnelle en Oncologie, INSERM, 91401 Orsay, France; 4 Institut Curie, PSL Research University, Radiation Oncology Department, Proton Therapy Centre, Centre Universitaire, 91898 Orsay, France; 5 INSERM U1196/UMR9187 CMIB, University Paris-Saclay, Institut Curie-Recherche, bât. 112, rue H. Becquerel, 91405 Orsay, France; 6 CNRS, Sorbonne Université, R2424, ABiMS platform, Station Biologique, 29680 Roscoff, France; 7 Radiotherapy Department, Cancer Centre François Baclesse, 14000 Caen, France; 8 SAPHYN/ARCHADE (Advanced Resource Centre for HADrontherapy in Europe), Cancer Centre François Baclesse, 14000 Caen, France

**Keywords:** Gene expression analysis, Non-coding RNAs, Transcriptomics, Cancer therapy

## Abstract

Proton therapy allows to avoid excess radiation dose on normal tissues. However, there are some limitations. Indeed, passive delivery of proton beams results in an increase in the lateral dose upstream of the tumor and active scanning leads to strong differences in dose delivery. This study aims to assess possible differences in the transcriptomic response of skin in C57BL/6 mice after TBI irradiation by active or passive proton beams at the dose of 6 Gy compared to unirradiated mice. In that purpose, total RNA was extracted from skin samples 3 months after irradiation and RNA-Seq was performed. Results showed that active and passive delivery lead to completely different transcription profiles. Indeed, 140 and 167 genes were differentially expressed after active and passive scanning compared to unirradiated, respectively, with only one common gene corresponding to RIKEN cDNA 9930021J03. Moreover, protein–protein interactions performed by STRING analysis showed that 31 and 25 genes are functionally related after active and passive delivery, respectively, with no common gene between both types of proton delivery. Analysis showed that active scanning led to the regulation of genes involved in skin development which was not the case with passive delivery. Moreover, 14 ncRNA were differentially regulated after active scanning against none for passive delivery. Active scanning led to 49 potential mRNA-ncRNA pairs with one ncRNA mainly involved, Gm44383 which is a miRNA. The 43 genes potentially regulated by the miRNA Gm44393 confirmed an important role of active scanning on skin keratin pathway. Our results demonstrated that there are differences in skin gene expression still 3 months after proton irradiation versus unirradiated mouse skin. And strong differences do exist in late skin gene expression between scattered or scanned proton beams. Further investigations are strongly needed to understand this discrepancy and to improve treatments by proton therapy.

## Introduction

Radiotherapy is a double-edge sword as it can be efficient on tumors but it can also alter normal tissues crossed by the radiations. Proton therapy (PT) should allow to avoid side effects on healthy tissues as the dose deposit is maximal at the end of the beam course under the form of a Bragg peak. Moreover, PT is often indicated for children cancers when organ at risk (OAR) are at proximity of the tumors. However, toxicities are still encountered after treatment by PT. Indeed, several studies on pediatric patients treated by PT for medulloblastoma^1^, brain tumors^2^, head and neck cancers^3^ and lung tumors^4^ have shown an increase in side effects compared to conventional radiotherapy, including Intensity-modulated radiotherapy.

These toxicities could be linked to the limitations of PT. Indeed, heterogeneity can occur in the lateral penumbra due to the variety of tissues encountered^5^. Moreover, to treat the entire volume of a tumor, Bragg peaks have to be added (SOBP, Spread-Out Bragg Peak) thus resulting in an increase in the dose received upstream of the tumor where plateau phases are also added^6^. In addition, PT can be delivered by two different techniques: passive scattering or active scanning. Passive beam delivery is the older technique: dose is delivered by a scattered proton beam which conforms to the tumor by collimators and compensator. This results in an increase in the lateral dose upstream of the tumor in normal tissues^7^ as shown in patients treated for breast cancer^8^. Active beam delivery allows a better conformation of the dose to the tumor^7^ but it leads to strong disparities in dose delivery in normal and tumor tissues in terms of: (1) dose rate (up to several Gy/s in the distal layer of the tumor); (2) duration (some seconds in the distal zone compared to several minutes in the proximal layer); and (3) fractionation (dose delivered at once for the most distal part compared to a lot of times in the proximal part)^9^. This can result in differences in biological responses in OAR localized near the tumor.

In the literature, there are few studies concerning effects of irradiation in the plateau phase before the Bragg peak of proton beams on transcription in normal tissues. Gridley et al. have shown an increase in gene expression of Prdx6 and Sod3 only after total body irradiation (TBI) proton irradiation in mice^10^. In the same manner, an increase in the transcription of NOX4 was observed by Chang et al. in hematopoietic stem cells after TBI proton mouse irradiation^11^. In rat eye exposed to proton beams, Mao et al*.* have shown an increase in the expression of genes involved in apoptosis and oxidative stress response^12^. In mouse brain, the expression of genes related to oxidative stress was also changed after protons compared to photons^13^. In an in vitro model of primary dermal fibroblasts, Nielsen et al. have shown that transcription of genes involved in inflammation and malignant transformation were differentially regulated by protons compared to photons^14^. And there are even less studies on gene expression after Pencil Beam Scanning (PBS) versus Double Scattering (DS) proton beams. Gridley et al. assessed 152 genes related to p53 and DNA damage pathways in human lung epithelial cells, only 2 genes were differentially expressed by a factor more than 2 in the plateau phase before the Bragg peak : BTG2 and SIAH1 concerning passive delivery, and only one : WT1 for active scanning^15^.

Conventional paradigm is that ionizing radiations mainly act via DNA damage^16^. More recent observations led to rethink this theory. Actually, epigenetic effects are one of the most relevant pathways with non-targeted effects (for review,^17^). Among epigenetic modifications, DNA methylation, histone modifications and non-coding RNAs (ncRNAs) modulations are the most important. Indeed, human genome code for 98% for ncDNA. Resulting ncRNAs, like lncRNAs and miRNA, can regulate mRNAs by inhibiting their translation or by inducing their degradation^18,19^. miRNAs levels were shown to change after irradiation in in vitro models^20–23^ and also in vivo^24–26^. They could play an essential role in cell death and cycle arrest^27^. Therefore, miRNAs are interesting targets to study as they could be implicated in radiation sensitivity^28–32^. Khan et al*.* showed that 2 Gy TBI proton irradiation of mice led to specific patterns of miRNAs according to the organ in brain, testis and liver^33^.

Our study aims at evaluating transcriptional responses after PBS versus DS proton beams. In this purpose, C57BL/6 which are able to develop side effects after irradiation, were total body irradiated in the plateau phase before the Bragg peak and skin samples were taken 3 months after irradiation to perform transcriptional analysis of total RNA, *i.e.* mRNA and ncRNA. Sequencing led to the differentially expressed genes (DEGs), STRING analysis revealed potential protein–protein interactions and analysis of GO-terms enrichments allowed to point out implicated pathways. Potential interactions between mRNAs of regulated genes and ncRNAs were also investigated.

## Materials and methods

### Animals

Ten weeks-old C57BL/6 mice were purchased at Charles River Laboratories (L’Arbresle, France). C57BL/6 mice had been specifically selected for genetic background. C57BL/6 are known to allow the development of late side effects after irradiation unlike other genetic backgrounds that preferentially develop early effects. To overcome the impact of the ovarian cycle and thus avoid a known sex effect in radiation-induced carcinogenesis, experiments were performed on male animals^34^. Mice were housed in a continuously controlled environment, with enrichment adapted to their species. Seven days were observed between the arrival of the mice and the application of the experimental procedures to allow them to acclimatize to their new environment and thus limit their stress.

### Ethics statement

All experimental procedures involving mice were conducted in compliance with the experimental research protocol approved by the ethics committee of the Institut Curie CEEA-IC #118 (authorization APAFiS# 27721-2020101612316744-v3 given by National Authority) and in accordance with the European Union Council (2010/63/UE) and ARRIVE guidelines for the use of laboratory animals.

### Total body irradiation with PBS or DS proton beams

**Figure 1:**
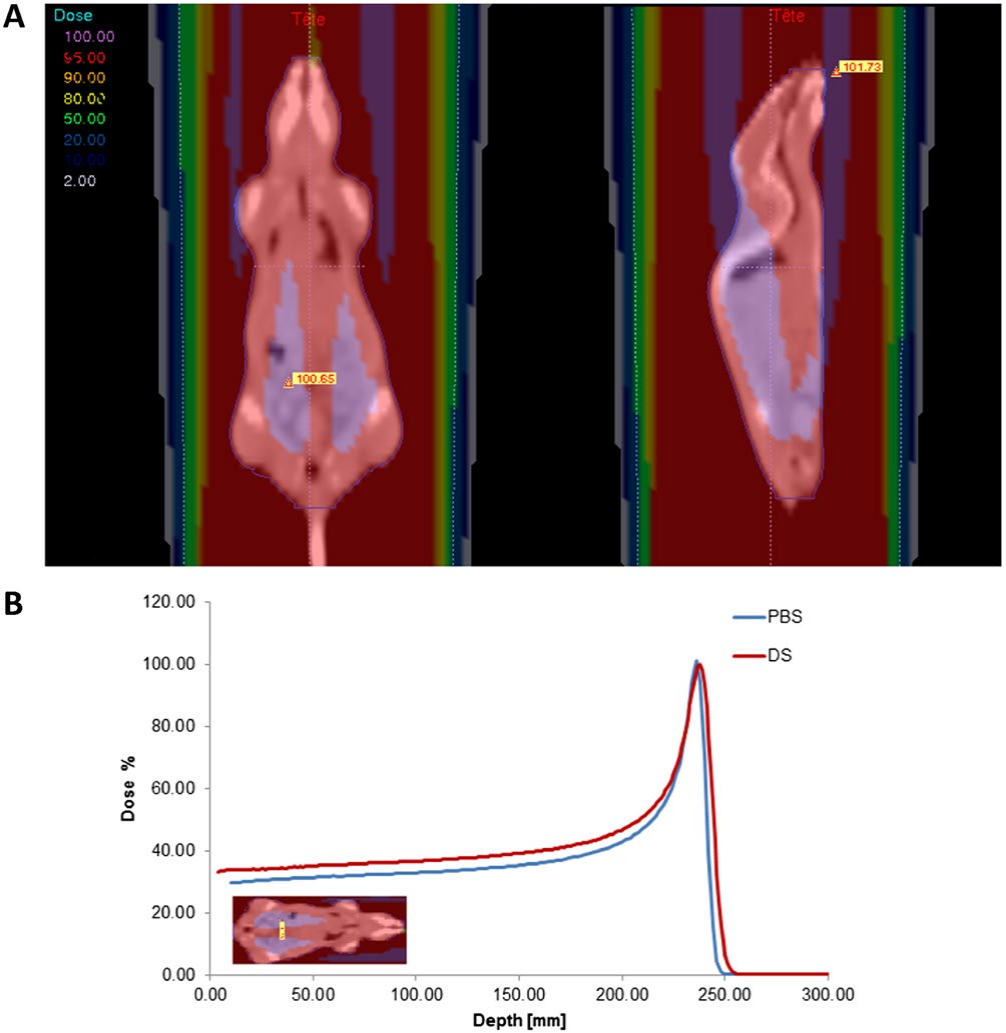
Dose distribution for murine experiments: (**A**) dose color wash using computed tomography of a mouse for the 5 cm diameter irradiation and double-scattered 190.6 MeV proton beam and (**B**) the corresponding depth dose distribution is also shown for both irradiation modes.

Irradiations were performed the same day for both cohorts at the Proton Therapy Center (CPO, Institut Curie, Orsay, France) using a C230 IBA accelerator providing proton beams either DS or PBS. Mice (n = 9 per condition) received a single sub-lethal dose of 6 Gy (physical dose) at an energy of 190.6 MeV in the plateau phase of the Bragg peak. For the IBA DS clinical machine, nozzle-specific settings are automatically chosen in order to achieve the desired field size, range, and modulation width. The range of the DS mode was then set to 23.9 g/cm^2^ and the modulation to the smallest value: this un-modulated beam was achieved by irradiating without rotating the modulator and placing the beam on the first thickness of the modulation wheel. The PBS energy (190.6 MeV) has been selected in order to ensure that both modes had the same range. The spot size at isocenter was approximately 4.5 mm, and a 5 × 5 cm^2^ field was created by scanning a single, narrow, monoenergetic proton-beam incident on the central axis with spots arranged in a uniform grid (same weight for all spots) with center-to-center spacing of 3 mm. Several studies have already made comparisons between these two modes^35,36^, and no significant differences were found for the LET_d_ (dose-averaged linear energy transfer) values between the two modes. In this way, radiation field was the smallest possible to ensure the whole body irradiation and radiation time and linear energy transfer were equivalent for both delivery modes. The mean dose rate used was the standard rate in conventional treatment settings: 6 Gy in 11 s for PBS (32 Gy/min) and 6 Gy in 2.5 min for DS (2.4 Gy/min). Each mouse was irradiated individually. The absolute dose per monitor unit was determined at the center of the field in the plateau region of the pseudo-monoenergetic 190.6 MeV pristine Bragg peak (at a measurement depth of 3 g cm^−2^) according to IAEA TRS-398 recommendations^37^. A plane parallel ionization chamber (PPC05, IBA dosimetry, Belgium), with a 9.9 mm diameter sensitive volume was used for absorbed dose-to-water measurements. The chamber was cross-calibrated under reference conditions at the isocenter against a Semiflex-type chamber calibrated under reference conditions in a ^60^Co beam at the French national metrology institute (CEA-LNHB), in terms of absorbed dose-to-water^37,38^. Polarity and recombination effects lower than 0.3% were found with this chamber for both modes, and a beam quality correction factor kQ of 1.028 was used. Dose distribution in the mouse is shown in Fig. 1 with a dose color wash using a CT (computed tomography) of a mouse with a 5 cm collimator and a 190.6 MeV Bragg peak (ISOGRAY, DOSISOFT, Cachan, France). Dose inhomogeneity is estimated at ± 5% by dose calculation (pencil beam scanning algorithm) within the mouse. Control mice followed the same course as irradiated mice. Mice were anesthetized with 4% isoflurane before being placed in prone position on a stage positioned in the irradiation field. The legs and tail of the mouse were taped with a flexible adhesive tape to prevent movement. The gas anesthesia was maintained with the mask throughout the irradiation, which will last a maximum of 15 min. Isoflurane was increased to 1.5–2% in 50% air room/50% oxygen. Mouse is detached after irradiation and placed in a clean cage during the awakening time.

### Follow-up and skin taking

Mice statute was monitored with caution during seven day after irradiation to detect any early signs of distress or ill-being. Mice were sacrificed 3 months after irradiation or when a weight loss greater than 20% of the initial weight was observed. When the mice were sacrificed, total exsanguination was performed to avoid the presence of blood in skin samples. In brief, mice were deeply anesthetized under 5% isoflurane throughout sampling. Rib cage was opened and then the blood was taken by direct puncture into the heart using a 25G needle followed by cervical dislocation after blood sampling. Then, skin from posterior legs (and other organs for another study) were collected.

### mRNA and ncRNA sequencing

Total RNAs from posterior leg skin were extracted from 36 different mice (9 per condition). Skin samples have been ground using a cryogenic grinder (SPEX SamplePrep LLC, Methucen, NJ). Obtained powder was put in 1 mL of TRIzol (Ambion, Life Technologies, Carlsbad, CA, USA) before solvent extraction performed according to manufacturer recommendations. RNA concentrations and quality (A260/A280 ratio) were assessed by spectrophotometry (NanoDrop 2000; Thermo Fisher Scientific, Waltham, MA, USA) and RNA integrity was evaluated (denaturing gel electrophoresis). The 9 RNA samples per condition were then pooled randomly by three. A twelve-sample stranded library preparation was performed using a NEBNext rRNA-depleted (HMR) kit (New England BioLabs, Ipswich, MA, USA). The sequencing of 100-pb paired-end reads was performed on an Illumina Novaseq 6000 Sequencer (Illumina, San Diego, CA, USA).

### RNAseq data analysis

The raw datasets for the twelve libraries were cleaned and trimmed with Trimmomatic v0.39: (using defaults parameters)^39^. Quantification for the abundances of transcripts was performed using pseudo-aligner Salmon v1.1.0^40^ against cDNA or ncRNA of *Mus musculus* GRCm38 database. Differential gene expression analyses were performed using TMM^41^ normalization counts by DEseq2 [Love, 2014] and edgeR^42^. Significant (*p*-value adj. < 0.05) differentially expressed genes (DEGs) (fold change ≥ 2) were analyzed. A STRING analysis (v11.0, https://string-db.org) was performed (minimum interaction score > 0.7) to reveal potential protein interaction encoded by DEGs and corresponding GO-terms enrichments^43^. For identifying ncRNA-mRNA coexpression only pairs of DEGs mRNA-lncRNA with a Pearson correlation coefficient above 0.98 between TMM expression level were kept. LncTar software^44^ was used to predict potential mRNA-ncRNA interaction (using ndG < -0.20). Putative mRNA-ncRNA interaction network was drawn with CYTOSCAPE (v2.8.3, www.cytoscape.org).

### Statistics

Body weights were analyzed by one-way ANOVA (normality and homogeneity of variances were previously checked). Differences were considered significant at *P* < 0.05. Differential expression analyses and other resulting analyses were conducted by comparing the expression levels of the transcribed between irradiated mice group and their respective non-irradiated control group.

## Results

### Follow-up of the mice after PBS or DS irradiation

**Figure 2:**
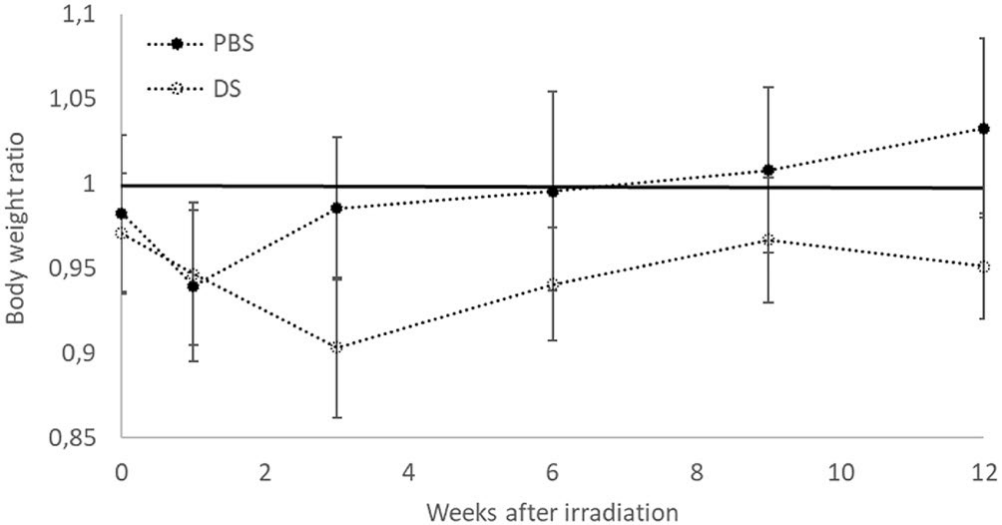
Mouse body weight ratio after PBS or DS 3 months after irradiation. Weight ratio was calculated as follows: irradiated mouse body weight divided by unirradiated mouse body weight. N = 9 in each group. Error bars represent standard error of the mean (SEM).

After irradiation, mouse body weight tends to decrease after PBS or DS irradiation before returning to the same level as unirradiated mice several weeks after irradiation (Fig. 2). When comparing PBS and DS irradiation, there is a non-significant trend to a lower weight after DS than after PBS. No mice died or were euthanized due to weight loss greater than 20% of the initial weight. The two control groups of non-irradiated mice showed no significant differences. Moreover, all mice presented hair depigmentation.

### Overview of the sequencing after PBS or DS irradiation

Table 1 provides an overview of sequencing metrics. Three replicates of pooled total RNAs from 3 different animal were sampled by experiment (DS and PBS) and by dose condition (0 and 6 Gy). In the end, 12 cDNA libraries were sequenced. Despite a lower number of reads in the 6 PBS proton beam samples, the remapping as well as the number of identifications was greater than DS proton beam samples testifying to a greater sample homogeneity within PBS proton beam experiment.

**Table 1:** Overview of sequencing, assembly and analysis.

	DS	PBS
Total number of bases sequenced	93,995,555,464	85,127,017,962
Reads	465,324,532	421,420,881
Remapping (mRNA + ncRNA)	74.35%	77.16%
mRNA transcripts identified	34,093	36,747
ncRNA transcripts identified	19,746	19,738
Number of coding DEGs	167	140
Number of non-coding DEGs	0	14
Number of putative interactions ncRNA-mRNA	0	49

### Quantitative analysis of the differentially expressed gene number after PBS or DS irradiation

**Figure 3:**
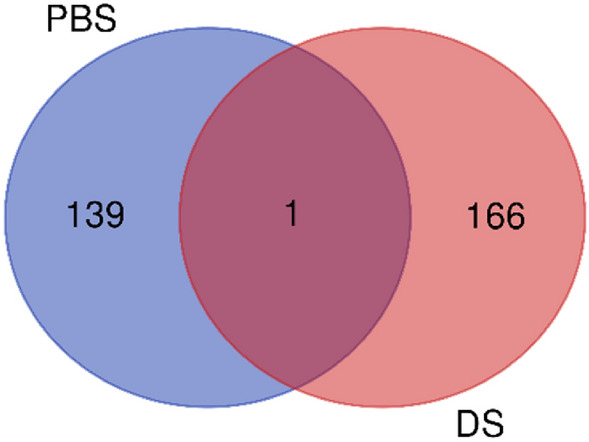
Distribution of differentially expressed genes between irradiated mice and their corresponding control group. Numbers corresponds to transcripts whose expression levels vary by more than a factor 2 and whose adjusted *P value* is less than 0.05.

Filtering of differentially expressed transcribed (|FC|> 2; padj < 0.05) showed that 140 and 167 genes were still regulated 3 months after the irradiation of mice with PBS and DS proton beam respectively (Fig. 3). Only ENSMUST0000175764.8 corresponding to RIKEN cDNA 9930021J03 gene is regulated by both types of irradiation.

### List of differentially expressed genes after PBS or DS irradiation

Supplementary Table S1 presents the complete list of mouse differentially expressed transcripts 3 months after irradiation with PBS proton beam and compared to non-irradiated mice group. A 128 transcripts were founded to be overexpressed and 12 downregulated compared to unirradiated controls. Only 4 genes showed two isoforms: Cux1, Padi3, Taf5l and Slc25a37. Keratin-related genes were highly represented: 17 transcripts coding for keratin and 21 for keratin associated proteins.

Supplementary Table S2 presents the complete list of mouse differentially expressed transcripts 3 months after irradiation with DS proton beam and compared to non-irradiated mouse group. A 110 transcripts were founded to be overexpressed and 57 downregulated compared to unirradiated controls. Twelve genes showed two isoforms: Casr, Casc4, Evi2, Hdx, Marveld2, Olfr1258, Retreg2, Serpina3m, Sla, Ubp1, Vmn1r220 and Wdr61.

Supplementary Table S3 presents the list of the 14 mouse differentially expressed non-coding transcripts 3 months after irradiation with DS proton beam and compared to non-irradiated mouse group. Among these 14 non-coding RNAs, all are upregulated compared to control group. Gm44383, Gm44393 and Gm44460 belong to the class of miRNA. No non-coding RNAs passed filtering after irradiation with DS proton beam (|FC|> 2, p-adj < 0.05); ending further analysis.

### Protein–protein interactions of regulated genes after PBS or DS irradiation

**Figure 4:**
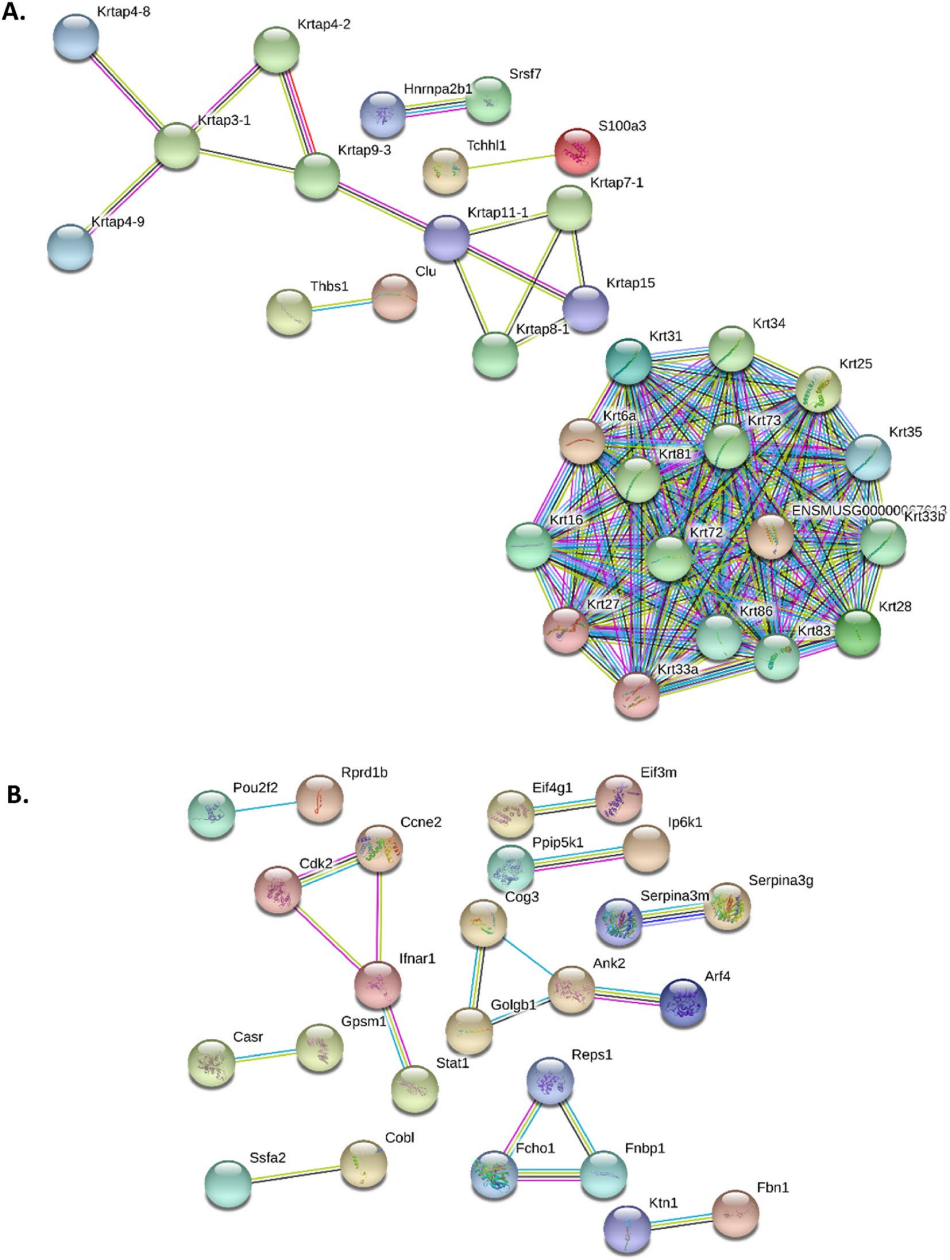
Protein–protein interaction of mouse skin genes regulated 3 months after (**A**) PBS or (**B**) DS irradiation (STRING analysis).

STRING protein–protein interaction analysis revealed that 31 of the 140 and 25 of the 167 genes regulated, after irradiation with PBS (Fig. 4A) and DS (Fig. 4B) proton beam respectively, were functionally related. However, none of the regulated genes were common between the two types of irradiation.

### GO-term enrichment of differentially expressed genes after PBS or DS irradiation

Analysis of GO-terms enrichments also confirmed the presence of two very different regulated gene profiles. While genes regulated after irradiation with PBS proton beam were centered on skin development and more precisely the formation of keratin (Table 2), those regulated after DS proton beam were less precise and localized mainly at the organelle level (Table 3).

**Table 2:** GO-terms enrichment of differentially expressed genes after PBS irradiation.

Biological process (GO)			
GO-term	Description	Count in gene set	False discovery rate
GO:0042633	Hair cycle	7 of 95	0.0032
GO:0008544	Epidermis development	10 of 237	0.0032
GO:0043588	Skin development	9 of 220	0.0035

Molecular function (GO)			
GO-term	Description	Count in gene set	False discovery rate
GO:0005198	structural molecule activity	20 of 546	2.40e−08

Cellular component (GO)			
GO-term	Description	Count in gene set	False discovery rate
GO:0005882	Intermediate filament	26 of 120	1.65e−29
GO:0045111	Intermediate filament Cytoskeleton	27 of 158	1.47e−28
GO:0099513	Polymeric cytoskeletal fiber	29 of 581	5.32e−17
GO:0099512	Supramolecular fiber	30 of 809	2.25e−14
GO:0045095	Keratin filament	11 of 43	3.95e−13
GO:0044430	Cytoskeletal part	31 of 1460	5.53e−09
GO:0005856	Cytoskeleton	34 of 1933	6.68e−08
GO:0043232	Intracellular non-membrane-bounded organelle	43 of 3809	9.94e−05
GO:0005903	Brush border	6 of 133	0.0035

**Table 3:** GO-terms enrichment of differentially expressed genes after DS irradiation.

Biological process (GO)			
GO-term	Description	Count in gene set	False discovery rate
–			

Molecular function (GO)			
GO-term	Description	Count in gene set	False discovery rate
GO:0005515	Protein binding	58 of 6456	0.0319
GO:0005488	Binding	83 of 10,884	0.0469

Cellular component (GO)			
GO-term	Description	Count in gene set	False discovery rate
GO:0044464	Cell part	101 of 14,017	0.0043
GO:0044424	Intracellular part	93 of 12,219	0.0043
GO:0043231	Intracellular membrane-bounded organelle	76 of 9088	0.0043
GO:0043229	Intracellular organelle	85 of 10,645	0.0043
GO:0043227	Membrane-bounded organelle	80 of 9775	0.0043
GO:0043226	Organelle	87 of 10,897	0.0043
GO:0005623	Cell	101 of 14,044	0.0043
GO:0005622	Intracellular	94 of 12,462	0.0043
GO:0097135	Cyclin E2-CDK2 complex	2 of 2	0.0084
GO:0005634	Nucleus	54 of 6086	0.0087
GO:0005737	Cytoplasm	76 of 9909	0.0192
GO:0044444	Cytoplasmic part	62 of 7673	0.0267
GO:0044425	Membrane part	50 of 5857	0.0349
GO:0016020	Membrane	60 of 7460	0.0349

### Potential interactions between regulated gene mRNAs and non-coding RNAs after PBS irradiation

No non-coding RNAs passed filtering after irradiation with DS proton beam. After PBS irradiation, the combined analysis of the correlation of expression levels and theoretical required hybridization energy leaded to identify 49 potential mRNA-ncRNA pairs (Table 4).

**Table 4:** Theoretical hybridization energy required to form ncRNA-mRNA pairs after PBS irradiation.

Query	Length_Query	Target	Length_Target	dG	ndG
Gm44393	138	Krtap1-4	593	− 19.08	− 0.2
Gm44393	138	Slc7a8	4084	− 20.67	− 0.2
Gm44393	138	Hoxc13	2461	− 19.52	− 0.2
Gm44393	138	Krtap1-5	1041	− 18.17	− 0.2
Gm44393	138	Krtap4-16	954	− 17.41	− 0.2
Gm44393	138	Krt31	1581	− 19.17	− 0.2
Gm44393	138	Krt34	1609	− 19.17	− 0.2
Gm44393	138	Krtap4-7	984	− 17.81	− 0.2
Gm44393	138	Krt28	1624	− 20.46	− 0.2
Gm44393	138	Krtap16-1	1888	− 18.65	− 0.2
Gm44393	138	Krtap4-9	1059	− 18.71	− 0.2
Gm44393	138	Capn12	3040	− 21.05	− 0.2
Gm44393	138	Dlx3	2607	− 21.23	− 0.2
Gm44393	138	Krtap4-2	974	− 18.20	− 0.2
Gm44393	138	Gm11938	663	− 20.76	− 0.2
Gm44393	138	Gm11562	817	− 20.77	− 0.2
Gm44393	138	Krtap2-4	828	− 20.77	− 0.2
Gm44393	138	Gm11937	396	− 20.77	− 0.2
Gm44393	138	Gprc5d	1317	− 14.67	− 0.2
Gm44393	138	Gjb2	2406	− 17.81	− 0.2
Gm44393	138	Krtap9-3	759	− 20.79	− 0.2
Gm44393	138	Pinlyp	843	− 19.13	− 0.2
Gm44393	138	Gm11567	1043	− 18.79	− 0.2
Gm44460	129	Ucp2	1116	− 18.97	− 0.2
Gm44393	138	Krt35	1722	− 20.46	− 0.2
Gm44460	129	Marcksl1	1605	− 19.99	− 0.2
Gm44393	138	Krtap3-2	1031	− 18.26	− 0.2
Gm44460	129	Gja1	3071	− 17.45	− 0.2
Gm44393	138	Krtap1-3	879	− 21.63	− 0.2
Gm44383	129	Tnc	7100	− 19.86	− 0.2
Gm44393	138	Dsg4	3478	− 24.15	− 0.2
Gm44393	138	Padi1	3754	− 20.22	− 0.2
Gm44393	138	Atp12a	3950	− 25.21	− 0.2
Gm44393	138	Padi3	3072	− 24.83	− 0.2
Gm44393	138	Cpm	5195	− 19.25	− 0.2
Gm44393	138	Krt27	1550	− 22.84	− 0.2
Gm44393	138	Tchh	5823	− 29.20	− 0.2
Gm44393	138	Krt33b	1581	− 19.68	− 0.2
Gm44393	138	S100a3	712	− 19.14	− 0.2
Gm44393	138	Fhod3	4956	− 30.56	− 0.2
Gm44460	129	Slc25a37	5927	− 23.27	− 0.3
Gm44393	138	Krt87	2278	− 57.09	− 0.4
Gm44393	138	Krt72	1953	− 60.81	− 0.5
Gm44393	138	Krt83	1757	− 60.84	− 0.5
Gm44393	138	Krt81	1843	− 61.96	− 0.5
Gm44393	138	Krt86	1963	− 61.96	− 0.5
Gm44393	138	Krt71	2186	− 61.62	− 0.5
Gm13032	2001	Padi1	3754	− 1344.53	− 0.7
Gm44393	138	Krt73	2168	− 93.22	− 0.7

**Figure 5:**
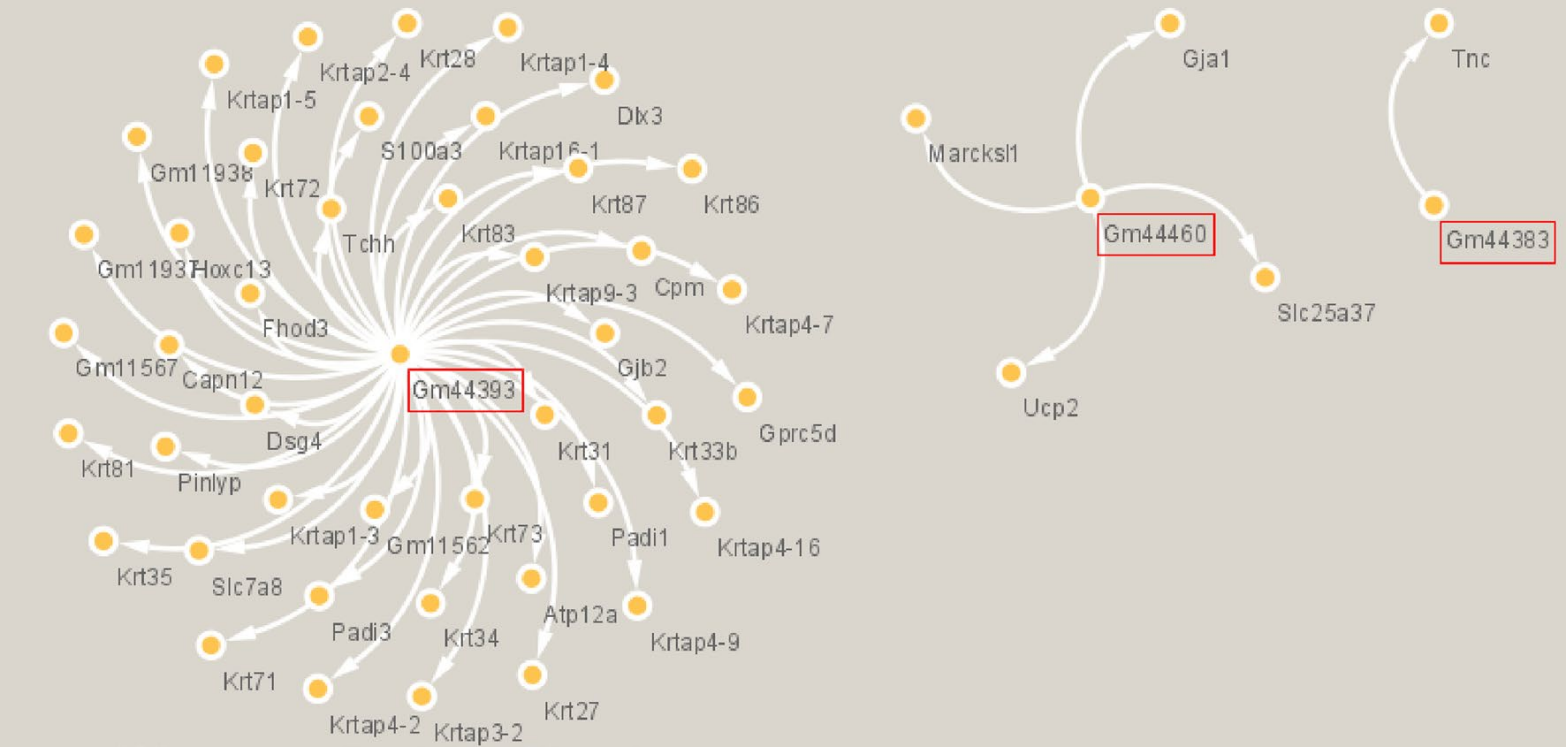
Representation of potential mRNA regulations by the 3 miRNAs identified after PBS irradiation (CYTOSCAPE analysis). Red rectangles represent the 3 miRNAs potentially involved in mRNA regulation.

Among the 14 ncRNAs differentially expressed, 3 ncRNAs (Gm44393, Gm44460 and Gm44383) were identified as potential mRNA regulators (Fig. 5). Gm44393 should be implicated in 43 of the 49 potentially regulation identified.

### Keratinization as a major actor of the late effects observed with PBS?

Table 5 shows that GO-terms enrichment analysis of the 43 genes potentially regulated by Gm44393 miRNA confirmed the impact of PBS irradiation on keratin formation pathway.

**Table 5:** GO-Terms Enrichment of putative Gm44393 linked DEGs.

Biological process (GO)			
GO-term	Description	Count in gene set	False discovery rate
GO:0042633	Hair cycle	5 of 95	0.0017
GO:1905867	Epididymis development	2 of 4	0.0092
GO:0043588	Skin development	5 of 220	0.0092
GO:0036414	Histone citrullination	2 of 5	0.0092
GO:0001942	Hair follicle development	4 of 84	0.0092
GO:0008544	Epidermis development	5 of 237	0.0109

Molecular function (GO)			
GO-term	Description	Count in gene set	False discovery rate
GO:0005198	Structural molecule activity	13 of 546	1.31e−08
GO:0004668	Protein-arginine deiminase activity	2 of 5	0.0062
GO:0005243	Gap junction channel activity	2 of 14	0.0176

Cellular component (GO)			
GO-term	Description	Count in gene set	False discovery rate
GO:0005882	Intermediate filament	16 of 120	2.49e-22
GO:0045095	Keratin filament	9 of 43	5.16e-14
GO:0099512	Supramolecular fiber	17 of 809	1.01e-11
GO:0044430	Cytoskeletal part	17 of 1460	5.53e-08
GO:0005856	Cytoskeleton	17 of 1933	2.95e-06
GO:0043232	Intracellular non-membrane-bounded organelle	18 of 3809	0.0059
GO:0005922	Connexin complex	2 of 20	0.0095

## Discussion

This study aims to assess possible differences in the transcriptomic response of skin in C57BL/6 mice after TBI irradiation in the plateau phase of the Bragg peak by active or passive proton beams at the sublethal dose of 6 Gy compared to unirradiated mice. Indeed, few studies have been performed on transcription after these 2 delivery techniques and even less on normal tissues. At the clinical level, Matsubara et al. have shown that beam scanning was not always favorable in patients treated for breast cancer by carbon ions but carbon ions and protons present completely different physical and biological properties^45^.

Concerning irradiation modalities, the energy (190.6 MeV) was chosen as it was the maximum energy possible with the DS system allowing large field dimensions to be obtained. Indeed, there are limitations in the combination of range, modulation and field size in the clinical DS mode. Moreover, this high energy allowed to have a dose that is as homogeneous as possible in the plateau region, at the entrance of the Bragg peak.

All irradiated mice (active and passive scanning) did not present significant change in their weight during the 3 months of breeding, which confirms that the chosen TBI sub-lethal dose of 6 Gy is adequate to see the appearance of late effects. Indeed, weight loss is a side effect widely described for a very long time in irradiated mice as TBI causes digestive system failure^46^. After a trend to a decrease after irradiation, mouse weight returned to basal level for both delivery modes. When comparing active and passive delivery, there was a non-significant trend to a lower weight gain from 3 weeks up to 3 months after the passive irradiation compared to the active one. This could be related to the difference in dose rate and delivery between PBS and DS. Indeed, PBS irradiation occurred by pulses and, as described in a previous work on Balb/c mice exposed to X-ray pulsed dose rate versus conventional radiotherapy, pulsed irradiation was shown to limit weight loss compared to conventional radiotherapy^47^. Further investigations are needed with a higher number of animals and with later times of observations as it could indicate that scattered beams are less well tolerated.

Concerning differential expression procedure and sample size, we followed recommendations as described by Schurch et al*.*^48^. Sequencing depth being also a critical point, 9 animals were included per condition, 50 M reads were generated per samples and gene differential expression calculation with DEseq2 (|FC|> 2, padj < 0.05) were performed. The study of DEGs 3 months after irradiation made it possible to identify impairs in gene expression. The total number of DEGs was relatively close: 140 and 167 genes were differentially expressed after active and passive scanning compared to unirradiated, respectively. Concerning the identified ncRNAs, no transcripts could be identified after passive scanning compared to unirradiated. This absence of regulators testifies to the proximity of the expression profiles between unirradiated mice and mice irradiated with passive scattering. We should think that major part of the DEGs would be common to the 2 types of irradiation. However, a single gene is commonly differentially expressed, i.e. RIKEN cDNA 9930021J03. In the literature, a study on human lung epithelial cells exposed to active or proton beams before the Bragg peak has also shown that transcription profiles were completely different between both techniques^15^.

This first clear difference in DEGs (Supplementary Tables S1 and S2) could have its origin in the physicochemical properties of the 2 types of irradiations. Indeed, if the protons are identical and the doses equivalent from a physical point of view, their mode of delivery is different. Passive scattering and active scanning lead to strong differences in dose delivery^7,9^. Secondarily, the DEGs after passive scattering did not seem to react to a particular stimulus as evidenced by the absence of GO-terms enrichment in the biological process category. These results highlight again the relatively close profiles between irradiated mice and unirradiated controls. In opposition, it is very clear that skin cells of mice irradiated with a proton beam actively scanned continue to respond to particular stimuli inducing an activation of the synthesis of 17 cytoskeletal and cuticular keratins of type I and II (Supplementary Tables S1 and S2) 3 months after the irradiation. These results should be waited as irradiation is known to induce acute keratin gene expression impair^49^. But we could expect 3 months after irradiation a return to basal state of keratinocyte with an expression of Keratin 5. Instead of that, Kr6 and Kr16 were overexpressed highlighting that keratinocyte activation was occurring and due to cutaneous late damage still under wounding^50^.

After analysis of potential interactions between DEGs transcripts and differentially expressed ncRNAs, 3 miRNAs were highlighted: Gm44393, Gm44460 and Gm44383 (Supplementary Table S3). Gm44393 seems to be of particular interest as it appears to interact with 43 of the 49 identified DEGs transcripts. The miRNA Gm44393 appears to target genes of keratinization pathway. Strategies based on miRNA expression modulation are promising tools to new treatment protocols. Jiang et al.^51^ have discovered a specific miRNA pattern expression in psoriatic epidermis. Indeed, miR-486-3p is not expressed allowing Keratin 17 protein overexpression and leading to the pathogenesis of psoriasis. miRNA Gm44393 activation specifically after active scanning proton beam irradiation is a step forward in fundamental knowledge of healthy tissue recovery and bring new perspectives on the modulation of PT cutaneous side effects.

To conclude, this study highlighted at the transcriptomic level great differences in skin response 3 months after irradiation. Profiles of DEGs were very distinct. On one hand, after passive proton beam, mRNAs expression was slightly different from control and no differences could be observed in ncRNAs. On the other hand, active scanning led to an overexpression of mRNAs related to keratin and putative miRNAs keratin regulators. Future investigations are strongly needed to explain the differences in biological responses observed between PBS and DS. Indeed, differences were also recently shown in terms of genotoxicity, oxidative stress and inflammation in various organs in total body irradiated C57BL/6 mice exposed to scattered versus scanned proton beams^52^. These findings pointed out the absolute need to adapt the PT protocols according to the type of scanning.

## Supplementary Information

Supplementary Information.
