# Publisher Correction: Differential normal skin transcriptomic response in total body irradiated mice exposed to scattered versus scanned proton beams

**DOI:** 10.1038/s41598-021-97156-z

**Published:** 2021-08-30

**Authors:** Alexandre Leduc, Samia Chaouni, Frédéric Pouzoulet, Ludovic De Marzi, Frédérique Megnin-Chanet, Erwan Corre, Dinu Stefan, Jean-Louis Habrand, François Sichel, Carine Laurent

**Affiliations:** 1 Normandie Univ, UNICAEN, UNIROUEN, ABTE-EA465114000, ToxEMAC, Cancer Centre François Baclesse, Caen, France; 2 Institut Curie, RadeXp Platform, centre universitaire, 91405 Orsay, France; 3 Institut Curie, PSL Research University, University Paris Saclay, Laboratoire d’Imagerie Translationnelle en Oncologie, INSERM, 91401 Orsay, France; 4 Institut Curie, PSL Research University, Radiation Oncology Department, Proton Therapy Centre, Centre Universitaire, 91898 Orsay, France; 5 INSERM U1196/UMR9187 CMIB, University Paris-Saclay, Institut Curie-Recherche, bât. 112, rue H. Becquerel, 91405 Orsay, France; 6 CNRS, Sorbonne Université, R2424, ABiMS platform, Station Biologique, Roscof, France; 7 Radiotherapy Department, Cancer Centre François Baclesse, 14000 Caen, France; 8 SAPHYN/ARCHADE (Advanced Resource Centre for HADrontherapy in Europe), Cancer Centre François Baclesse, 14000 Caen, France

Correction to: *Scientific Reports* 10.1038/s41598-021-85394-0, published online 12 March 2021

The original version of this Article contained a repeated error, where the unit of measurement “MeV” was incorrectly given as “meV.”

The original Article has been corrected.

