# Supplementary material for: Differential normal skin transcriptomic response in total body irradiated mice exposed to scattered versus scanned proton beams
Source: Sci Rep. 2021 Mar 12;11:5876. doi: 10.1038/s41598-021-85394-0 (PMC7955113; doi:10.1038/s41598-021-85394-0)
Supplement: Supplementary file 1 — Supplementary Information. [file 41598_2021_85394_MOESM1_ESM.docx]

**Supplementary data**

**Differential normal skin transcriptomic response in total body irradiated mice exposed to scattered *versus* scanned proton beams**

Alexandre Leduc^1^, Samia Chaouni^1^, Frédéric Pouzoulet^2^, Ludovic De Marzi^3^, Frédérique Megnin-Chanet^4^, Erwan Corre^5^, Dinu Stefan^1,6^, Jean-Louis Habrand^1,6^, François Sichel^1^, Carine Laurent^1,7,*^

^1^ Normandie Univ, UNICAEN, UNIROUEN, ABTE-EA4651, ToxEMAC, 14000, Caen, France

^2^ Institut Curie, RadeXp Platform, centre universitaire, 91405 Orsay, France

^3^ Centre de protonthérapie d'Orsay, Institut Curie, PSL Research university, campus universitaire, bâtiment 101, 91898 Orsay, France; Institut Curie, university Paris Saclay, PSL Research university, Inserm U 1021-CNRS UMR 3347, Orsay, France

^4^ INSERM U1196/UMR9187 CMIB, University Paris-Saclay, Institut Curie-Recherche, bât. 112, rue H. Becquerel, 91405 Orsay, France

^5^ CNRS, Sorbonne Université, FR2424, ABiMS platform, Station Biologique, 29680 Roscoff, France

^6^ Radiotherapy Department, Cancer Centre François Baclesse, 14000, Caen, France

^7^ SAPHYN/ARCHADE (Advanced Resource Centre for HADrontherapy in Europe), Cancer Centre François Baclesse, 14000, Caen, France

^*^ Correspondence: carine.laurent@unicaen.fr

**Keywords:** protontherapy; Double Scattering; Pencil Beam Scanning; side effects; skin; transcriptomic; mRNA; ncRNA

**Supplementary Table S1. List of differentially expressed genes 3 months after PBS irradiation.**

|  | | |  |  |
| --- | --- | --- | --- | --- |
| Upregulated genes | | | | |
| **Transcript stable ID version** | **Gene name** | **Gene description** | **log2FC** | **padj** |
| ENSMUST00000007340.3 | Atp12a | ATPase, H+/K+ transporting, nongastric, alpha polypeptide | 1,35 | 1,1E-02 |
| ENSMUST00000173433.7 | Baz1a | bromodomain adjacent to zinc finger domain 1A | 1,33 | 3,3E-05 |
| ENSMUST00000170054.8 | Baz2a | bromodomain adjacent to zinc finger domain, 2A | 10,98 | 1,5E-14 |
| ENSMUST00000048052.6 | Calhm4 | calcium homeostasis modulator family member 4 | 2,75 | 3,0E-04 |
| ENSMUST00000066880.5 | Capn12 | calpain 12 | 2,07 | 2,0E-03 |
| ENSMUST00000020399.5 | Cpm | carboxypeptidase M | 1,42 | 1,7E-02 |
| ENSMUST00000169576.7 | Cnot2 | CCR4-NOT transcription complex, subunit 2 | 1,18 | 5,6E-04 |
| ENSMUST00000028780.3 | Chac1 | ChaC, cation transport regulator 1 | 1,88 | 4,0E-03 |
| ENSMUST00000048665.7 | Chd9 | chromodomain helicase DNA binding protein 9 | 1,14 | 4,4E-02 |
| ENSMUST00000022616.13 | Clu | clusterin | 1,03 | 3,6E-02 |
| ENSMUST00000176778.7 | Cux1 | cut-like homeobox 1 | 1,63 | 1,2E-03 |
| ENSMUST00000175975.8 | Cux1 | cut-like homeobox 1 | 1,27 | 6,4E-03 |
| ENSMUST00000122919.7 | Ccnl1 | cyclin L1 | 1,28 | 5,9E-03 |
| ENSMUST00000085691.10 | Dmkn | dermokine | 9,87 | 7,0E-12 |
| ENSMUST00000019426.4 | Dsg4 | desmoglein 4 | 2,91 | 1,6E-05 |
| ENSMUST00000092768.6 | Dlx3 | distal-less homeobox 3 | 2,33 | 9,1E-04 |
| ENSMUST00000020118.4 | Dusp6 | dual specificity phosphatase 6 | 1,01 | 2,2E-03 |
| ENSMUST00000225200.1 | Egr3 | early growth response 3 | 1,20 | 5,9E-03 |
| ENSMUST00000049053.8 | Fam168a | family with sequence similarity 168, member A | 1,06 | 1,4E-04 |
| ENSMUST00000234834.1 | Fhod3 | formin homology 2 domain containing 3 | 3,40 | 1,9E-05 |
| ENSMUST00000111922.1 | Gprc5d | G protein-coupled receptor, family C, group 5, member D | 4,08 | 6,2E-06 |
| ENSMUST00000068581.8 | Gja1 | gap junction protein, alpha 1 | 1,67 | 2,4E-03 |
| ENSMUST00000055698.7 | Gjb2 | gap junction protein, beta 2 | 4,40 | 1,3E-06 |
| ENSMUST00000159985.1 | Hephl1 | hephaestin-like 1 | 1,11 | 1,8E-02 |
| ENSMUST00000090002.9 | Hnrnpa2b1 | heterogeneous nuclear ribonucleoprotein A2/B1 | 1,38 | 1,4E-03 |
| ENSMUST00000001700.6 | Hoxc13 | homeobox C13 | 1,51 | 4,1E-02 |
| ENSMUST00000113453.8 | Hopx | HOP homeobox | 1,06 | 5,2E-03 |
| ENSMUST00000007280.8 | Krt16 | keratin 16 | 1,13 | 8,5E-04 |
| ENSMUST00000038004.2 | Krt25 | keratin 25 | 5,50 | 1,5E-07 |
| ENSMUST00000017732.2 | Krt27 | keratin 27 | 5,26 | 5,2E-08 |
| ENSMUST00000006963.2 | Krt28 | keratin 28 | 3,77 | 6,9E-09 |
| ENSMUST00000007318.1 | Krt31 | keratin 31 | 5,16 | 4,2E-06 |
| ENSMUST00000018399.2 | Krt33a | keratin 33A | 5,45 | 8,0E-07 |
| ENSMUST00000073890.3 | Krt33b | keratin 33B | 5,05 | 2,6E-05 |
| ENSMUST00000056362.2 | Krt34 | keratin 34 | 4,98 | 1,3E-04 |
| ENSMUST00000103127.3 | Krt35 | keratin 35 | 7,89 | 1,7E-03 |
| ENSMUST00000023788.7 | Krt6a | keratin 6A | 1,13 | 1,7E-17 |
| ENSMUST00000023710.5 | Krt71 | keratin 71 | 5,78 | 1,3E-08 |
| ENSMUST00000071104.5 | Krt72 | keratin 72 | 4,58 | 5,2E-14 |
| ENSMUST00000063292.7 | Krt73 | keratin 73 | 4,04 | 3,4E-04 |
| ENSMUST00000061185.7 | Krt81 | keratin 81 | 5,16 | 9,4E-06 |
| ENSMUST00000023718.8 | Krt83 | keratin 83 | 5,76 | 1,2E-06 |
| ENSMUST00000088049.4 | Krt86 | keratin 86 | 5,08 | 2,9E-05 |
| ENSMUST00000081945.4 | Krt87 | keratin 87 | 4,73 | 4,0E-05 |
| ENSMUST00000171542.1 | Krtap11-1 | keratin associated protein 11-1 | 5,82 | 3,6E-06 |
| ENSMUST00000104930.1 | Krtap1-3 | keratin associated protein 1-3 | 5,71 | 6,7E-10 |
| ENSMUST00000189774.1 | Krtap14 | keratin associated protein 14 | 5,92 | 2,6E-07 |
| ENSMUST00000100479.2 | Krtap1-4 | keratin associated protein 1-4 | 4,82 | 7,4E-07 |
| ENSMUST00000187823.1 | Krtap15 | keratin associated protein 15 | 6,52 | 4,1E-06 |
| ENSMUST00000054532.4 | Krtap1-5 | keratin associated protein 1-5 | 5,89 | 8,8E-06 |
| ENSMUST00000105050.3 | Krtap16-1 | keratin associated protein 16-1 | 3,43 | 2,6E-03 |
| ENSMUST00000179707.2 | Krtap16-3 | keratin associated protein 16-3 | 4,70 | 1,7E-04 |
| ENSMUST00000074926.5 | Krtap2-4 | keratin associated protein 2-4 | 5,48 | 9,1E-05 |
| ENSMUST00000055502.4 | Krtap3-1 | keratin associated protein 3-1 | 6,16 | 9,1E-06 |
| ENSMUST00000092699.2 | Krtap3-2 | keratin associated protein 3-2 | 3,35 | 2,9E-02 |
| ENSMUST00000081007.6 | Krtap4-1 | keratin associated protein 4-1 | 5,80 | 2,4E-04 |
| ENSMUST00000107437.1 | Krtap4-16 | keratin associated protein 4-16 | 5,09 | 5,5E-04 |
| ENSMUST00000058987.4 | Krtap4-2 | keratin associated protein 4-2 | 4,00 | 9,1E-05 |
| ENSMUST00000100476.2 | Krtap4-6 | keratin associated protein 4-6 | 5,26 | 2,0E-08 |
| ENSMUST00000055121.6 | Krtap4-7 | keratin associated protein 4-7 | 5,43 | 1,3E-04 |
| ENSMUST00000107439.1 | Krtap4-8 | keratin associated protein 4-8 | 4,49 | 3,2E-07 |
| ENSMUST00000105059.3 | Krtap4-9 | keratin associated protein 4-9 | 3,68 | 1,0E-04 |
| ENSMUST00000056118.3 | Krtap7-1 | keratin associated protein 7-1 | 6,88 | 3,5E-08 |
| ENSMUST00000072280.4 | Krtap8-1 | keratin associated protein 8-1 | 6,49 | 2,4E-07 |
| ENSMUST00000062683.2 | Krtap9-3 | keratin associated protein 9-3 | 6,01 | 1,3E-09 |
| ENSMUST00000081496.5 | Ltbp3 | latent transforming growth factor beta binding protein 3 | 1,96 | 3,2E-03 |
| ENSMUST00000021607.8 | Lgmn | legumain | 1,42 | 1,3E-02 |
| ENSMUST00000046122.10 | Lap3 | leucine aminopeptidase 3 | 1,14 | 2,5E-02 |
| ENSMUST00000064606.7 | Lrrc15 | leucine rich repeat containing 15 | 2,76 | 2,1E-02 |
| ENSMUST00000116273.8 | Kdm1a | lysine (K)-specific demethylase 1A | 1,32 | 1,2E-04 |
| ENSMUST00000070658.15 | Mgrn1 | mahogunin, ring finger 1 | 1,09 | 3,5E-05 |
| ENSMUST00000062356.6 | Marcksl1 | MARCKS-like 1 | 1,19 | 4,4E-02 |
| ENSMUST00000037991.11 | Mical2 | microtubule associated monooxygenase, calponin and LIM domain containing 2 | 1,18 | 2,0E-03 |
| ENSMUST00000120259.7 | Map3k5 | mitogen-activated protein kinase kinase kinase 5 | 1,41 | 4,5E-03 |
| ENSMUST00000194069.5 | Mbnl1 | muscleblind like splicing factor 1 | 1,50 | 2,9E-05 |
| ENSMUST00000071935.6 | Myo9b | myosin IXb | 1,29 | 2,3E-09 |
| ENSMUST00000167856.7 | Myo18a | myosin XVIIIA | 1,22 | 1,9E-02 |
| ENSMUST00000071065.7 | Nfil3 | nuclear factor, interleukin 3, regulated | 1,01 | 5,2E-03 |
| ENSMUST00000030025.9 | Nr4a3 | nuclear receptor subfamily 4, group A, member 3 | 1,88 | 2,1E-02 |
| ENSMUST00000171737.2 | Odc1 | ornithine decarboxylase, structural 1 | 1,11 | 7,8E-06 |
| ENSMUST00000023698.11 | Paxbp1 | PAX3 and PAX7 binding protein 1 | 1,27 | 3,3E-02 |
| ENSMUST00000026378.3 | Padi1 | peptidyl arginine deiminase, type I | 2,81 | 6,5E-04 |
| ENSMUST00000026377.8 | Padi3 | peptidyl arginine deiminase, type III | 6,89 | 3,7E-04 |
| ENSMUST00000172098.1 | Padi3 | peptidyl arginine deiminase, type III | 1,68 | 8,5E-03 |
| ENSMUST00000051226.7 | Pfkm | phosphofructokinase, muscle | 1,29 | 3,4E-05 |
| ENSMUST00000011776.7 | Pinlyp | phospholipase A2 inhibitor and LY6/PLAUR domain containing | 3,36 | 1,8E-06 |
| ENSMUST00000101376.2 | Plb1 | phospholipase B1 | 1,39 | 3,0E-02 |
| ENSMUST00000222485.1 | Pitrm1 | pitrilysin metallepetidase 1 | 1,12 | 1,9E-02 |
| ENSMUST00000054449.13 | Plec | plectin | 12,04 | 5,0E-19 |
| ENSMUST00000092694.3 | Gm11559 | predicted gene 11559 | 2,68 | 1,2E-03 |
| ENSMUST00000073853.2 | Gm11562 | predicted gene 11562 | 5,70 | 4,7E-05 |
| ENSMUST00000092695.4 | Gm11563 | predicted gene 11563 | 4,81 | 7,4E-07 |
| ENSMUST00000078442.3 | Gm11567 | predicted gene 11567 | 4,11 | 7,2E-06 |
| ENSMUST00000107440.1 | Gm11595 | predicted gene 11595 | 4,54 | 1,8E-07 |
| ENSMUST00000076478.1 | Gm11937 | predicted gene 11937 | 5,16 | 4,6E-02 |
| ENSMUST00000072306.3 | Gm11938 | predicted gene 11938 | 5,45 | 6,2E-06 |
| ENSMUST00000230067.1 | Gm49425 | predicted gene, 49425 | 5,51 | 2,3E-06 |
| ENSMUST00000070284.3 | Prr9 | proline rich 9 | 3,39 | 5,5E-04 |
| ENSMUST00000036248.12 | Pmepa1 | prostate transmembrane protein, androgen induced 1 | 1,09 | 2,6E-02 |
| ENSMUST00000025273.8 | Psors1c2 | psoriasis susceptibility 1 candidate 2 (human) | 3,84 | 4,5E-03 |
| ENSMUST00000086801.6 | Rfx2 | regulatory factor X, 2 (influences HLA class II expression) | 1,18 | 1,9E-02 |
| ENSMUST00000175764.8 | 9930021J03Rik | RIKEN cDNA 9930021J03 gene | 1,03 | 7,4E-04 |
| ENSMUST00000098098.8 | Rnf38 | ring finger protein 38 | 1,23 | 1,2E-03 |
| ENSMUST00000009679.10 | Rnmt | RNA (guanine-7-) methyltransferase | 1,22 | 2,4E-02 |
| ENSMUST00000059647.11 | Rbm12 | RNA binding motif protein 12 | 1,66 | 4,2E-02 |
| ENSMUST00000001047.7 | S100a3 | S100 calcium binding protein A3 | 5,17 | 3,6E-05 |
| ENSMUST00000063417.10 | Srsf7 | serine and arginine-rich splicing factor 7 | 1,40 | 5,2E-07 |
| ENSMUST00000226785.1 | Styx | serine/threonine/tyrosine interaction protein | 1,26 | 3,9E-03 |
| ENSMUST00000222714.1 | Sipa1l1 | signal-induced proliferation-associated 1 like 1 | 10,32 | 1,7E-13 |
| ENSMUST00000184497.1 | Slc25a37 | solute carrier family 25, member 37 | 1,12 | 1,7E-02 |
| ENSMUST00000037064.4 | Slc25a37 | solute carrier family 25, member 37 | 1,09 | 7,1E-03 |
| ENSMUST00000022787.7 | Slc7a8 | solute carrier family 7 (cationic amino acid transporter, y+ system), member 8 | 2,23 | 2,7E-05 |
| ENSMUST00000110030.9 | Snx5 | sorting nexin 5 | 1,78 | 6,6E-03 |
| ENSMUST00000093039.5 | Taf5l | TATA-box binding protein associated factor 5 like | 1,44 | 2,7E-02 |
| ENSMUST00000213235.1 | Taf1d | TATA-box binding protein associated factor, RNA polymerase I, D | 1,23 | 4,0E-03 |
| ENSMUST00000142577.1 | Txlna | taxilin alpha | 1,39 | 2,9E-02 |
| ENSMUST00000089024.12 | Tcp1 | t-complex protein 1 | 1,30 | 6,1E-03 |
| ENSMUST00000107377.9 | Tnc | tenascin C | 1,69 | 2,6E-03 |
| ENSMUST00000098603.7 | Tet2 | tet methylcytosine dioxygenase 2 | 11,23 | 3,9E-15 |
| ENSMUST00000039559.8 | Thbs1 | thrombospondin 1 | 1,19 | 2,9E-05 |
| ENSMUST00000064257.5 | Tchh | trichohyalin | 5,17 | 2,1E-08 |
| ENSMUST00000029516.2 | Tchhl1 | trichohyalin-like 1 | 3,21 | 2,7E-05 |
| ENSMUST00000207748.1 | Ucp2 | uncoupling protein 2 (mitochondrial, proton carrier) | 1,53 | 1,0E-02 |
| ENSMUST00000042503.8 | Usp6nl | USP6 N-terminal like | 1,18 | 3,8E-02 |
| ENSMUST00000233808.1 | Vmn1r226 | vomeronasal 1 receptor 226 | 10,60 | 3,7E-13 |
| ENSMUST00000111635.2 | Xirp1 | xin actin-binding repeat containing 1 | 1,51 | 2,6E-02 |
| ENSMUST00000066617.11 | Zkscan1 | zinc finger with KRAB and SCAN domains 1 | 9,71 | 1,4E-10 |
| ENSMUST00000036176.14 | - | - | 1,94 | 3,6E-05 |
|  |  |  |  |  |
| Downregulated genes | | | | |
| ENSMUST00000112604.7 | Iqsec2 | IQ motif and Sec7 domain 2 | -1,01 | 3,4E-02 |
| ENSMUST00000165628.8 | Taf5l | TATA-box binding protein associated factor 5 like | -1,05 | 1,1E-02 |
| ENSMUST00000114299.7 | Flna | filamin, alpha | -1,11 | 1,4E-04 |
| ENSMUST00000189017.7 | Fat1 | FAT atypical cadherin 1 | -1,14 | 1,9E-02 |
| ENSMUST00000166646.1 | Abcc4 | ATP-binding cassette, sub-family C (CFTR/MRP), member 4 | -1,17 | 2,5E-02 |
| ENSMUST00000112435.8 | Ep400 | E1A binding protein p400 | -1,20 | 3,8E-04 |
| ENSMUST00000105527.1 | Tnfaip3 | tumor necrosis factor, alpha-induced protein 3 | -1,21 | 2,7E-02 |
| ENSMUST00000031583.14 | Acacb | acetyl-Coenzyme A carboxylase beta | -1,31 | 4,3E-02 |
| ENSMUST00000132151.7 | Fsd1l | fibronectin type III and SPRY domain containing 1-like | -1,81 | 2,2E-02 |
| ENSMUST00000165978.2 | Tecpr2 | tectonin beta-propeller repeat containing 2 | -1,89 | 2,7E-05 |
| ENSMUST00000215340.1 | Olfr536 | olfactory receptor 536 | -10,09 | 2,1E-13 |
| ENSMUST00000107272.5 | - | - | -10,18 | 7,1E-06 |
| Log2FC : log2 Fold change. |  |  |  |  |

**Supplementary Table S2. List of differentially expressed genes 3 months after DS irradiation.**

|  | | |  |  |
| --- | --- | --- | --- | --- |
| Upregulated genes | | | | |
| **Transcript stable ID version** | **Gene name** | **Gene description** | **log2FC** | **padj** |
| ENSMUST00000030221.2 | Hacd4 | 3-hydroxyacyl-CoA dehydratase 4 | 6,62 | 3,4E-02 |
| ENSMUST00000113059.7 | Adamts7 | a disintegrin-like and metallopeptidase (reprolysin type) with thrombospondin type 1 motif, 7 | 4,56 | 3,9E-02 |
| ENSMUST00000106824.7 | Ap4b1 | adaptor-related protein complex AP-4, beta 1 | 7,29 | 2,3E-05 |
| ENSMUST00000020984.8 | Adcy3 | adenylate cyclase 3 | 8,13 | 6,3E-08 |
| ENSMUST00000179758.7 | Adtrp | androgen dependent TFPI regulating protein | 7,78 | 6,7E-07 |
| ENSMUST00000025483.10 | Nars | asparaginyl-tRNA synthetase | 8,87 | 6,1E-07 |
| ENSMUST00000176192.2 | Atpaf1 | ATP synthase mitochondrial F1 complex assembly factor 1 | 7,42 | 1,5E-05 |
| ENSMUST00000112550.7 | Baz2b | bromodomain adjacent to zinc finger domain, 2B | 1,52 | 2,0E-03 |
| ENSMUST00000063597.13 | Casr | calcium-sensing receptor | 7,93 | 3,2E-06 |
| ENSMUST00000078752.9 | Casc4 | cancer susceptibility candidate 4 | 1,71 | 1,6E-03 |
| ENSMUST00000164088.7 | Cnot2 | CCR4-NOT transcription complex, subunit 2 | 9,15 | 2,2E-10 |
| ENSMUST00000167086.1 | Cd101 | CD101 antigen | 6,99 | 1,4E-02 |
| ENSMUST00000114548.7 | Cadm1 | cell adhesion molecule 1 | 1,67 | 3,7E-02 |
| ENSMUST00000203646.2 | Clasp1 | CLIP associating protein 1 | 2,18 | 1,1E-02 |
| ENSMUST00000220227.1 | Coq10a | coenzyme Q10A | 7,43 | 3,6E-03 |
| ENSMUST00000027766.12 | Coq8a | coenzyme Q8A | 8,78 | 1,6E-08 |
| ENSMUST00000237125.1 | Coro1b | coronin, actin binding protein 1B | 6,63 | 2,0E-02 |
| ENSMUST00000029866.15 | Ccne2 | cyclin E2 | 1,86 | 1,9E-03 |
| ENSMUST00000069937.10 | Cdkl2 | cyclin-dependent kinase-like 2 (CDC2-related kinase) | 8,17 | 1,3E-06 |
| ENSMUST00000229524.1 | Cyhr1 | cysteine and histidine rich 1 | 7,16 | 1,5E-03 |
| ENSMUST00000197489.4 | Dcun1d1 | DCN1, defective in cullin neddylation 1, domain containing 1 (S. cerevisiae) | 7,64 | 5,1E-06 |
| ENSMUST00000139324.7 | Donson | downstream neighbor of SON | 3,57 | 3,2E-03 |
| ENSMUST00000093983.10 | Evi2 | ecotropic viral integration site 2 | 9,57 | 1,6E-11 |
| ENSMUST00000113783.7 | Eda | ectodysplasin-A | 8,41 | 1,3E-07 |
| ENSMUST00000132376.1 | Ech1 | enoyl coenzyme A hydratase 1, peroxisomal | 7,09 | 1,2E-04 |
| ENSMUST00000111110.2 | Eif3m | eukaryotic translation initiation factor 3, subunit M | 5,61 | 5,3E-04 |
| ENSMUST00000141972.7 | C87436 | expressed sequence C87436 | 6,90 | 2,2E-02 |
| ENSMUST00000060807.11 | Fam83h | family with sequence similarity 83, member H | 2,25 | 4,9E-03 |
| ENSMUST00000148272.1 | Fbn1 | fibrillin 1 | 7,20 | 6,2E-05 |
| ENSMUST00000138581.2 | Fnbp1 | formin binding protein 1 | 7,48 | 1,9E-05 |
| ENSMUST00000172405.7 | Ggnbp2 | gametogenetin binding protein 2 | 8,78 | 3,9E-08 |
| ENSMUST00000114812.8 | Golgb1 | golgi autoantigen, golgin subfamily b, macrogolgin 1 | 1,12 | 2,4E-02 |
| ENSMUST00000078616.11 | Gpsm1 | G-protein signalling modulator 1 (AGS3-like, C. elegans) | 3,13 | 3,1E-02 |
| ENSMUST00000193597.1 | Gucy1b1 | guanylate cyclase 1, soluble, beta 1 | 7,34 | 3,5E-03 |
| ENSMUST00000137865.7 | Hp1bp3 | heterochromatin protein 1, binding protein 3 | 7,11 | 1,0E-04 |
| ENSMUST00000072357.13 | Hk1 | hexokinase 1 | 8,14 | 6,0E-06 |
| ENSMUST00000113422.8 | Hdx | highly divergent homeobox | 9,16 | 9,4E-11 |
| ENSMUST00000177158.1 | Ip6k1 | inositol hexaphosphate kinase 1 | 6,94 | 6,6E-04 |
| ENSMUST00000117748.7 | Ifnar1 | interferon (alpha and beta) receptor 1 | 7,68 | 1,3E-06 |
| ENSMUST00000111785.8 | Itprid2 | ITPR interacting domain containing 2 | 1,28 | 4,7E-07 |
| ENSMUST00000173949.1 | Jmjd1c | jumonji domain containing 1C | 7,58 | 2,2E-05 |
| ENSMUST00000187262.6 | Ktn1 | kinectin 1 | 8,70 | 3,0E-09 |
| ENSMUST00000118583.7 | Ltbp4 | latent transforming growth factor beta binding protein 4 | 1,68 | 5,9E-06 |
| ENSMUST00000126573.7 | Lrp8 | low density lipoprotein receptor-related protein 8, apolipoprotein e receptor | 7,65 | 9,8E-05 |
| ENSMUST00000058771.12 | Lysmd4 | LysM, putative peptidoglycan-binding, domain containing 4 | 6,56 | 3,1E-02 |
| ENSMUST00000168772.1 | Marveld2 | MARVEL (membrane-associating) domain containing 2 | 6,58 | 4,1E-02 |
| ENSMUST00000106857.7 | Mier1 | MEIR1 treanscription regulator | 3,21 | 3,5E-02 |
| ENSMUST00000025580.9 | Ms4a6b | membrane-spanning 4-domains, subfamily A, member 6B | 6,79 | 4,6E-03 |
| ENSMUST00000100214.9 | Miga2 | mitoguardin 2 | 1,60 | 4,6E-03 |
| ENSMUST00000186442.6 | Mndal | myeloid nuclear differentiation antigen like | 7,13 | 1,9E-04 |
| ENSMUST00000102505.9 | Myo1c | myosin IC | 1,15 | 1,7E-02 |
| ENSMUST00000151273.7 | Gnptab | N-acetylglucosamine-1-phosphate transferase, alpha and beta subunits | 7,57 | 5,5E-05 |
| ENSMUST00000121778.7 | Ndufb5 | NADH:ubiquinone oxidoreductase subunit B5 | 7,28 | 2,9E-04 |
| ENSMUST00000095947.10 | Nrap | nebulin-related anchoring protein | 4,85 | 6,1E-04 |
| ENSMUST00000177020.1 | Nptn | neuroplastin | 7,38 | 1,3E-04 |
| ENSMUST00000225583.1 | Ntrk2 | neurotrophic tyrosine kinase, receptor, type 2 | 4,46 | 5,1E-03 |
| ENSMUST00000153353.8 | Nop56 | NOP56 ribonucleoprotein | 7,18 | 1,7E-04 |
| ENSMUST00000188507.6 | Nemp2 | nuclear envelope integral membrane protein 2 | 7,90 | 5,8E-07 |
| ENSMUST00000213789.2 | Olfr1065 | olfactory receptor 1065 | 9,41 | 3,0E-11 |
| ENSMUST00000102609.2 | Olfr1258 | olfactory receptor 1258 | 9,25 | 5,9E-11 |
| ENSMUST00000173286.7 | Otud4 | OTU domain containing 4 | 1,11 | 1,0E-02 |
| ENSMUST00000141055.1 | Otub2 | OTU domain, ubiquitin aldehyde binding 2 | 1,32 | 1,8E-02 |
| ENSMUST00000150545.7 | Pdrg1 | p53 and DNA damage regulated 1 | 7,34 | 7,5E-05 |
| ENSMUST00000086123.10 | Pitpnm2 | phosphatidylinositol transfer protein, membrane-associated 2 | 9,88 | 2,3E-12 |
| ENSMUST00000004955.13 | Prpsap2 | phosphoribosyl pyrophosphate synthetase-associated protein 2 | 8,02 | 9,7E-06 |
| ENSMUST00000107044.9 | Plekhb1 | pleckstrin homology domain containing, family B (evectins) member 1 | 7,22 | 8,0E-05 |
| ENSMUST00000185333.1 | Kcna6 | potassium voltage-gated channel, shaker-related, subfamily, member 6 | 7,39 | 7,5E-04 |
| ENSMUST00000120959.7 | Gm14434 | predicted gene 14434 | 7,84 | 1,3E-06 |
| ENSMUST00000168733.8 | Gm8206 | predicted gene 8206 | 9,04 | 2,8E-10 |
| ENSMUST00000184766.7 | Prorp | protein only RNase P catalytic subunit | 7,29 | 2,5E-03 |
| ENSMUST00000156263.1 | Rad23b | RAD23 homolog B, nucleotide excision repair protein | 7,10 | 1,5E-04 |
| ENSMUST00000154718.7 | Reps1 | RalBP1 associated Eps domain containing protein | 1,85 | 3,6E-02 |
| ENSMUST00000105782.1 | Rsc1a1 | regulatory solute carrier protein, family 1, member 1 | 8,97 | 2,8E-09 |
| ENSMUST00000190240.6 | Retreg2 | reticulophagy regulator family member 2 | 1,77 | 9,4E-03 |
| ENSMUST00000003741.15 | Rps6ka1 | ribosomal protein S6 kinase polypeptide 1 | 3,69 | 7,9E-05 |
| ENSMUST00000175764.8 | 9930021J03Rik | RIKEN cDNA 9930021J03 gene | 1,16 | 4,8E-03 |
| ENSMUST00000070705.5 | Rlim | ring finger protein, LIM domain interacting | 1,19 | 2,0E-02 |
| ENSMUST00000101078.11 | Serpina3m | serine (or cysteine) peptidase inhibitor, clade A, member 3M | 6,96 | 5,3E-04 |
| ENSMUST00000102544.8 | Srsf10 | serine and arginine-rich splicing factor 10 | 7,48 | 3,1E-05 |
| ENSMUST00000186857.6 | Stat1 | signal transducer and activator of transcription 1 | 8,28 | 5,7E-08 |
| ENSMUST00000204768.1 | Snrpg | small nuclear ribonucleoprotein polypeptide G | 6,98 | 1,1E-03 |
| ENSMUST00000027368.5 | Slc11a1 | solute carrier family 11 (proton-coupled divalent metal ion transporters), member 1 | 7,17 | 1,9E-04 |
| ENSMUST00000184169.7 | Slc25a12 | solute carrier family 25 (mitochondrial carrier, Aralar), member 12 | 8,06 | 4,7E-06 |
| ENSMUST00000096514.10 | Slc35a2 | solute carrier family 35 (UDP-galactose transporter), member A2 | 7,14 | 9,9E-04 |
| ENSMUST00000212270.1 | Slc38a7 | solute carrier family 38, member 7 | 9,31 | 5,6E-11 |
| ENSMUST00000098816.9 | Slc7a2 | solute carrier family 7 (cationic amino acid transporter, y+ system), member 2 | 6,99 | 6,6E-04 |
| ENSMUST00000100496.4 | Spryd7 | SPRY domain containing 7 | 7,74 | 5,3E-05 |
| ENSMUST00000100572.9 | Sla | src-like adaptor | 7,29 | 4,2E-05 |
| ENSMUST00000233897.1 | Sod2 | superoxide dismutase 2, mitochondrial | 7,76 | 1,0E-06 |
| ENSMUST00000099131.10 | Smarcc2 | SWI/SNF related, matrix associated, actin dependent regulator of chromatin, subfamily c, member 2 | 1,82 | 8,7E-04 |
| ENSMUST00000136189.7 | Tbc1d14 | TBC1 domain family, member 14 | 1,48 | 3,3E-03 |
| ENSMUST00000002391.14 | Tm9sf1 | transmembrane 9 superfamily member 1 | 9,10 | 1,3E-08 |
| ENSMUST00000100806.5 | Tmem254b | transmembrane protein 254b | 4,24 | 1,1E-02 |
| ENSMUST00000153292.7 | Trmt2a | TRM2 tRNA methyltransferase 2A | 6,82 | 4,6E-03 |
| ENSMUST00000154004.7 | Uty | ubiquitously transcribed tetratricopeptide repeat gene, Y chromosome | 1,01 | 5,3E-04 |
| ENSMUST00000162313.7 | Unc5cl | unc-5 family C-terminal like | 2,05 | 3,4E-02 |
| ENSMUST00000009885.13 | Ubp1 | upstream binding protein 1 | 7,78 | 6,7E-05 |
| ENSMUST00000226786.1 | Vmn1r198 | vomeronasal 1 receptor 198 | 9,66 | 3,0E-11 |
| ENSMUST00000227950.1 | Vmn1r220 | vomeronasal 1 receptor 220 | 9,36 | 5,6E-11 |
| ENSMUST00000051822.12 | Wdr61 | WD repeat domain 61 | 8,52 | 2,9E-08 |
| ENSMUST00000162541.7 | Wnk1 | WNK lysine deficient protein kinase 1 | 8,25 | 7,9E-07 |
| ENSMUST00000168247.1 | Zfp110 | zinc finger protein 110 | 7,27 | 3,2E-05 |
| ENSMUST00000182122.7 | Zfp236 | zinc finger protein 236 | 8,89 | 6,5E-08 |
| ENSMUST00000072292.11 | Zfp280c | zinc finger protein 280C | 8,75 | 1,3E-07 |
| ENSMUST00000209061.1 | Zfp36 | zinc finger protein 36 | 1,44 | 1,4E-02 |
| ENSMUST00000234956.1 | Zfp598 | zinc finger protein 598 | 7,52 | 6,8E-05 |
| ENSMUST00000088102.11 | Zfx | zinc finger protein X-linked | 10,38 | 5,8E-13 |
| ENSMUST00000049168.7 | - | - | 9,26 | 3,6E-09 |
| ENSMUST00000022593.6 | - | - | 8,01 | 5,9E-06 |
| ENSMUST00000109261.9 | - | - | 1,56 | 1,7E-09 |
|  |  |  |  |  |
| Downregulated genes | | | | |
| ENSMUST00000187485.6 | Adgrb1 | adhesion G protein-coupled receptor B1 | -7,22 | 4,9E-05 |
| ENSMUST00000053106.6 | Amigo2 | adhesion molecule with Ig like domain 2 | -6,84 | 3,1E-02 |
| ENSMUST00000166075.7 | Arf4 | ADP-ribosylation factor 4 | -1,68 | 4,3E-03 |
| ENSMUST00000182064.8 | Ank2 | ankyrin 2, brain | -9,02 | 3,1E-09 |
| ENSMUST00000182627.7 | Atp13a4 | ATPase type 13A4 | -7,20 | 1,9E-04 |
| ENSMUST00000005830.14 | Bcas2 | breast carcinoma amplified sequence 2 | -6,92 | 7,2E-04 |
| ENSMUST00000114847.8 | Casr | calcium-sensing receptor | -7,83 | 6,7E-07 |
| ENSMUST00000110586.9 | Casc4 | cancer susceptibility candidate 4 | -9,67 | 3,0E-11 |
| ENSMUST00000128557.2 | Champ1 | chromosome alignment maintaining phosphoprotein 1 | -8,35 | 4,1E-05 |
| ENSMUST00000109651.8 | Cobl | cordon-bleu WH2 repeat | -8,46 | 9,0E-08 |
| ENSMUST00000026416.14 | Cdk2 | cyclin-dependent kinase 2 | -1,16 | 4,9E-02 |
| ENSMUST00000112302.7 | Ctps2 | cytidine 5'-triphosphate synthase 2 | -1,74 | 1,7E-02 |
| ENSMUST00000110625.7 | Ppip5k1 | diphosphoinositol pentakisphosphate kinase 1 | -8,24 | 1,9E-05 |
| ENSMUST00000088976.11 | Dnmt3b | DNA methyltransferase 3B | -7,97 | 1,2E-05 |
| ENSMUST00000170422.3 | Evi2 | ecotropic viral integration site 2 | -9,59 | 1,3E-11 |
| ENSMUST00000115457.7 | Eif4g1 | eukaryotic translation initiation factor 4, gamma 1 | -9,27 | 6,7E-08 |
| ENSMUST00000097643.9 | AW554918 | expressed sequence AW554918 | -8,69 | 8,0E-07 |
| ENSMUST00000031891.14 | Fam131b | family with sequence similarity 131, member B | -7,95 | 1,0E-05 |
| ENSMUST00000136640.7 | Fcho1 | FCH domain only 1 | -6,65 | 3,4E-02 |
| ENSMUST00000178276.7 | Fgfr1 | fibroblast growth factor receptor 1 | -8,86 | 1,8E-08 |
| ENSMUST00000238192.1 | Hsf2bp | heat shock transcription factor 2 binding protein | -2,92 | 4,1E-02 |
| ENSMUST00000038472.6 | Hdx | highly divergent homeobox | -9,22 | 2,3E-10 |
| ENSMUST00000114130.8 | Ikbkg | inhibitor of kappaB kinase gamma | -8,88 | 5,7E-08 |
| ENSMUST00000225754.1 | Marveld2 | MARVEL (membrane-associating) domain containing 2 | -8,00 | 2,5E-05 |
| ENSMUST00000213720.2 | Olfr1258 | olfactory receptor 1258 | -9,56 | 3,0E-11 |
| ENSMUST00000215861.1 | Olfr1392 | olfactory receptor 1392 | -7,44 | 1,6E-03 |
| ENSMUST00000214192.1 | Olfr824 | olfactory receptor 824 | -2,50 | 2,2E-04 |
| ENSMUST00000194663.5 | Ptch1 | patched 1 | -1,61 | 2,0E-04 |
| ENSMUST00000230948.1 | Phf20l1 | PHD finger protein 20-like 1 | -9,09 | 1,4E-08 |
| ENSMUST00000200097.4 | Ptbp2 | polypyrimidine tract binding protein 2 | -1,59 | 3,1E-02 |
| ENSMUST00000108415.9 | Pou2f2 | POU domain, class 2, transcription factor 2 | -2,60 | 1,3E-07 |
| ENSMUST00000165289.7 | Gm3298 | predicted gene 3298 | -9,32 | 5,6E-11 |
| ENSMUST00000055576.11 | Pcsk6 | proprotein convertase subtilisin/kexin type 6 | -1,04 | 1,5E-02 |
| ENSMUST00000029180.13 | Rprd1b | regulation of nuclear pre-mRNA domain containing 1B | -8,87 | 1,0E-08 |
| ENSMUST00000203471.2 | Rfc1 | replication factor C (activator 1) 1 | -8,00 | 1,8E-07 |
| ENSMUST00000186037.6 | Retreg2 | reticulophagy regulator family member 2 | -8,45 | 3,9E-08 |
| ENSMUST00000149147.7 | Rnf157 | ring finger protein 157 | -1,06 | 3,5E-03 |
| ENSMUST00000168797.1 | Serpina3m | serine (or cysteine) peptidase inhibitor, clade A, member 3M | -7,04 | 5,4E-04 |
| ENSMUST00000081799.5 | Sgsm2 | small G protein signaling modulator 2 | -1,63 | 2,0E-02 |
| ENSMUST00000237371.1 | Slc29a2 | solute carrier family 29 (nucleoside transporters), member 2 | -6,88 | 2,6E-03 |
| ENSMUST00000055087.6 | Syk | spleen tyrosine kinase | -1,35 | 3,4E-02 |
| ENSMUST00000164163.7 | Sla | src-like adaptor | -4,30 | 2,5E-03 |
| ENSMUST00000154059.7 | St7 | suppression of tumorigenicity 7 | -8,21 | 3,1E-07 |
| ENSMUST00000132325.9 | Syt6 | synaptotagmin VI | -7,32 | 7,3E-05 |
| ENSMUST00000224480.1 | Tut7 | terminal uridylyl transferase 7 | -8,24 | 6,9E-07 |
| ENSMUST00000225139.1 | Tasor | transcription activation suppressor | -7,78 | 2,6E-04 |
| ENSMUST00000024833.12 | Tmprss3 | transmembrane protease, serine 3 | -7,82 | 4,9E-06 |
| ENSMUST00000110163.7 | Tmem230 | transmembrane protein 230 | -7,90 | 6,7E-06 |
| ENSMUST00000080336.3 | Tmem268 | transmembrane protein 268 | -8,56 | 1,4E-08 |
| ENSMUST00000225936.1 | Tpbpa | trophoblast specific protein alpha | -1,08 | 9,8E-03 |
| ENSMUST00000084885.11 | Ubp1 | upstream binding protein 1 | -7,85 | 2,9E-04 |
| ENSMUST00000227685.1 | Vmn1r199 | vomeronasal 1 receptor 199 | -2,48 | 3,8E-04 |
| ENSMUST00000226651.1 | Vmn1r220 | vomeronasal 1 receptor 220 | -10,35 | 1,6E-13 |
| ENSMUST00000228498.1 | Vmn1r34 | vomeronasal 1 receptor 34 | -8,81 | 5,1E-06 |
| ENSMUST00000121204.7 | Wdr61 | WD repeat domain 61 | -4,67 | 5,5E-03 |
| ENSMUST00000077485.10 | Zfp12 | zinc finger protein 12 | -8,93 | 7,0E-10 |
| ENSMUST00000146552.7 | - | - | -7,89 | 3,2E-06 |
| Log2FC : log2 Fold change. |  |  |  |  |

**Supplementary Table S3. List of differentially expressed non-coding RNA 3 months after PBS irradiation.**

|  | | | |  |  |
| --- | --- | --- | --- | --- | --- |
| **Transcript stable ID version** | **Gene name** | **Gene description** | **Type** | **log2FC** | **padj** |
| ENSMUST00000136820.1 | Gm11571 | predicted gene 11571 | Antisense | 1,80 | 1,61E-02 |
| ENSMUST00000153899.1 | Gm11655 | predicted gene 11655 | Antisense | 1,39 | 4,44E-02 |
| ENSMUST00000148335.1 | Gm15444 | predicted gene 15444 | Antisense | 1,98 | 2,25E-03 |
| ENSMUST00000218235.1 | Gm47767 | predicted gene 47767 | Antisense | 1,36 | 1,26E-03 |
| ENSMUST00000229164.1 | Gm49428 | predicted gene 49428 | Antisense | 1,19 | 7,27E-09 |
| ENSMUST00000226076.1 | Gm35110 | predicted gene 35110 | Bidirectional promoter lncRNA | 1,87 | 1,69E-02 |
| ENSMUST00000195343.1 | Gm10857 | predicted gene 10857 | LincRNA | 2,26 | 2,51E-03 |
| ENSMUST00000213219.1 | Gm48462 | predicted gene 48462 | LincRNA | 7,74 | 2,53E-06 |
| ENSMUST00000233522.1 | Gm49890 | predicted gene 49890 | LincRNA | 1,71 | 3,13E-02 |
| ENSMUST00000198940.1 | Gm44383 | predicted gene 44383 | MiRNA | 1,22 | 1,46E-02 |
| ENSMUST00000197418.1 | Gm44393 | predicted gene 44393 | MiRNA | 6,63 | 6,11E-04 |
| ENSMUST00000196179.1 | Gm44460 | predicted gene 44460 | MiRNA | 1,54 | 2,25E-03 |
| ENSMUST00000151848.1 | Gm13032 | predicted gene 13032 | Processed transcript | 2,45 | 1,09E-04 |
| ENSMUST00000162163.7 | Gas-5 | growth arrest specific 5 | Retained intron | 1,18 | 4,57E-02 |
| Log2FC : log2 Fold change. |  |  |  |  |  |
